# Bioinformatic Tools for the Analysis and Prediction of ncRNA Interactions

**DOI:** 10.3390/ijms222111397

**Published:** 2021-10-22

**Authors:** Andrés Rincón-Riveros, Duvan Morales, Josefa Antonia Rodríguez, Victoria E. Villegas, Liliana López-Kleine

**Affiliations:** 1 Bioinformatics and Systems Biology Group, Universidad Nacional de Colombia, Bogotá 111221, Colombia; warinconr@unal.edu.co; 2 Centro de Investigaciones en Microbiología y Biotecnología-UR (CIMBIUR), Facultad de Ciencias Naturales, Universidad del Rosario, Bogotá 111221, Colombia; duvan.morales@urosario.edu.co; 3 Grupo de Investigación en Biología del Cáncer, Instituto Nacional de Cancerología, Bogotá 111221, Colombia; jrodriguez@cancer.gov.co; 4 Department of Statistics, Faculty of Science, Universidad Nacional de Colombia, Bogotá 111221, Colombia

**Keywords:** genomics, transcriptome, ncRNA, lncRNA, interactome, bioinformatics, gene regulatory networks

## Abstract

Noncoding RNAs (ncRNAs) play prominent roles in the regulation of gene expression via their interactions with other biological molecules such as proteins and nucleic acids. Although much of our knowledge about how these ncRNAs operate in different biological processes has been obtained from experimental findings, computational biology can also clearly substantially boost this knowledge by suggesting possible novel interactions of these ncRNAs with other molecules. Computational predictions are thus used as an alternative source of new insights through a process of mutual enrichment because the information obtained through experiments continuously feeds through into computational methods. The results of these predictions in turn shed light on possible interactions that are subsequently validated experimentally. This review describes the latest advances in databases, bioinformatic tools, and new in silico strategies that allow the establishment or prediction of biological interactions of ncRNAs, particularly miRNAs and lncRNAs. The ncRNA species described in this work have a special emphasis on those found in humans, but information on ncRNA of other species is also included.

## 1. Introduction

The set of RNA molecules expressed in a cell or tissue is known as a transcriptome [1]. More than 90% of the human genome is transcribed, and less than 2% are protein-coding genes. This means that most transcribed genes produce noncoding RNAs (ncRNAs), the functions of which are mainly classified as either housekeeping or regulatory noncoding RNAs [2,3,4,5]. These ncRNAs have been shown to play leading roles in important biological processes such as the regulation of gene expression, which have the potential to maintain homeostasis, and a disruption of their functions can generate pathological processes [6,7]. These roles are performed via interactions between these ncRNAs and biological molecules such as proteins and nucleic acids, including both DNA and other types of RNA [8,9,10].

ncRNAs have been demonstrated to function in the nucleus and cytoplasm, interacting directly with genes or their products [11,12,13,14]. In the nucleus, ncRNAs have been shown to participate in processes that impact transcription, either as epigenetic regulators through the control of chromatin remodeling, or as mediators between transcription factors and gene promoters. Additionally in the nucleus, they can directly bind mRNAs and modulate splicing events through interaction with splicing factors, which allows the selection of one isoform of an mRNA over another. They serve as a poly(A) tail for those RNAs that fulfill their function in the cytoplasm and need this tail to reach the cytoplasm and not be degraded; they stabilize or destabilize coding or regulatory RNAs; and they even modulate passage through nuclear pores [12,15,16,17,18,19,20,21,22]. In the cytoplasm, ncRNAs participate in the regulation of various biological processes by interacting with other ncRNAs, mRNAs, or proteins [14,23,24].

Another interesting scenario, where interactions between heterogeneous molecules play an important role, is the case of RNA–protein interactions in the formation of cellular condensates [25]. Cell condensates are non-membranous assemblies (e.g., nucleolus, stress granules, P granules, nuclear speckles) where biomolecules are concentrated in the cell and are regularly formed by phase separation [26,27].

Although the understanding of cellular condensates is not completely clear, recent advances have shown that a network of intermolecular interactions with chemical and physical properties coexists. For example, as a result of some studies, RNAs present in the condensate have a modulating effect on its boundaries and behavior, binding to RBP and other proteins. These effects are given by the multiple characteristics of the RNA sequences (identity, length, and modification) and their environment (RNA structure, RNA–RNA interactions, and RNA–protein interactions) [28,29]. More about this topic is in a recent paper by Wiedner and Giudice [30].

Our knowledge of the roles of ncRNAs in biological processes that impact human health has largely been obtained from experimental findings [31,32,33,34]. However, computational biology can significantly boost this knowledge, providing insights into the possible interactions of these ncRNAs with other molecules, which can subsequently be verified experimentally [35,36,37]. Hence, computational predictions are an alternative approach that allows the expansion of knowledge through a process of mutual enrichment; this is because the information that is obtained experimentally continuously feeds into computational methods, and, in turn, the results of these predictions suggest possible interactions that can subsequently be validated through laboratory experiments using in vitro and/or in vivo models [31,33,34].

## 2. ncRNAs with Regulatory Functions

Noncoding RNA (ncRNA) genes are those genes that produce transcripts or functional RNAs that, unlike messenger RNAs, are not translated into proteins. Only a few years ago these transcripts were considered “dark matter” in the genome, but now they play leading roles in the regulation of biological processes [33,38].

**Figure 1:**
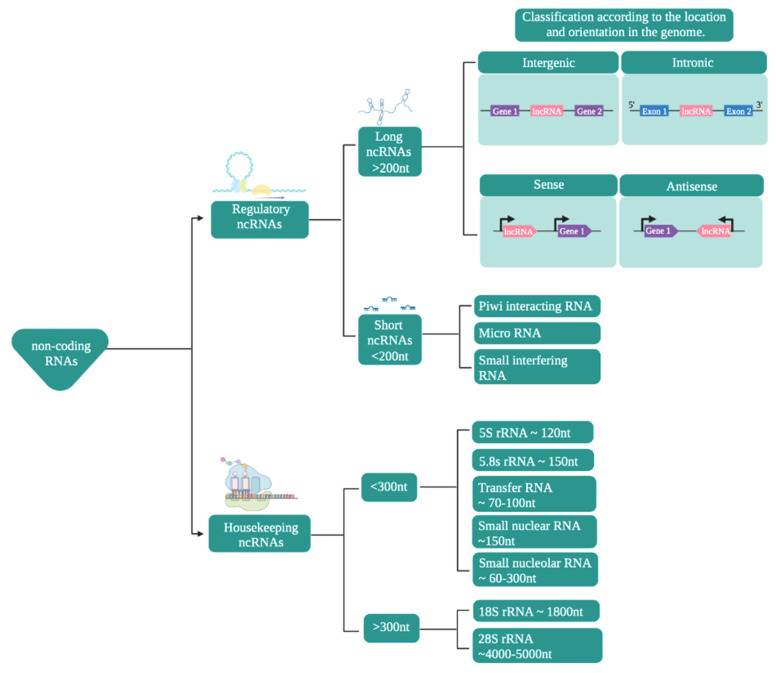
Classification of noncoding RNAs. The scheme presents the classification of noncoding RNAs according to their function, size, and location/orientation in the genome.

In terms of their functions, ncRNAs are mainly classified (Figure 1) into housekeeping and regulatory noncoding RNAs [39,40,41,42,43,44]. This review mainly focuses on ncRNAs that participate in the modulation of gene expression, by a variety of mechanisms [45,46].

Regulatory ncRNAs (Figure 1) are classified according to their size. ncRNAs of less than 200 nucleotides are known as small ncRNAs, while those larger than that are known as long noncoding RNAs (lncRNAs) [47,48,49,50].

The small ncRNAs include microRNAs (miRNAs), small interfering RNAs (siRNAs), and Piwi-interacting RNAs (piRNAs), among which miRNAs have become particularly prominent in research as they play important roles in the regulation of numerous cellular processes, making them potential treatment targets or biomarkers [51,52,53]. Mature miRNAs are between 19 and 23 nucleotides long and are transcribed by RNA polymerase II [44,54]. siRNAs, with a size of between 18 and 30 nucleotides, are the most diverse members of this group and can be of endogenous or exogenous origin. They can also regulate expression at the transcriptional and post-transcriptional levels [55,56].

lncRNAs are defined as transcripts that are at least 200 nucleotides in length [9,57]. They share some characteristics with mRNAs, such as both having exons and introns (although lncRNAs have fewer than mRNAs), most being generated by RNA Pol II, as well as a large proportion of them have a polyadenylated tail at their 3′ end and a methylated cap at their 5′ end [58,59]. These are located in intergenic regions or between exons [38,60] and have the ability to fold in on themselves and adopt various secondary and tertiary structures that maintain similar functions in evolutionarily distant species [61]. Regarding the expression levels of lncRNAs, they are very low, with variable expression patterns depending on the tissue, stage of development, or physiological or pathological state [62,63].

### 2.1. Regulation of the Gene Expression of ncRNAs through Their Interactions with Other Biological Molecules

**Figure 2:**
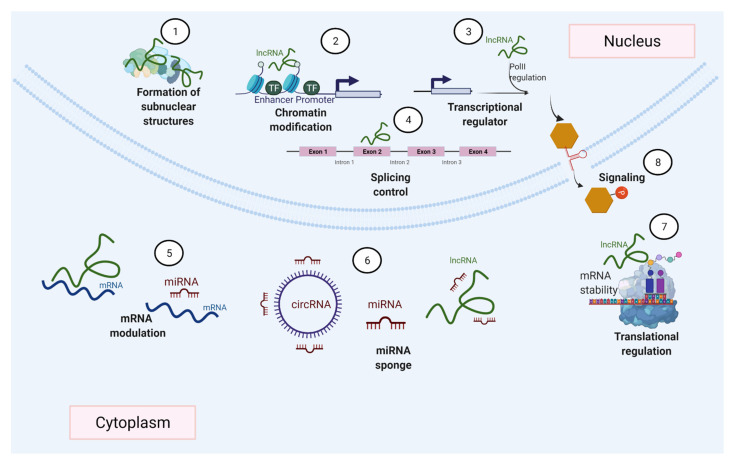
ncRNAs can regulate gene expression by diverse mechanisms. ncRNAs participate in the formation of nuclear bodies (1), gene transcription (2–3), modulate splicing events (4), regulate mRNA by degradation or stabilization (5), act as miRNA sponges (6), and ncRNAs can also be involved in the control of transcription (7) and cell signaling (8).

**Figure 3:**
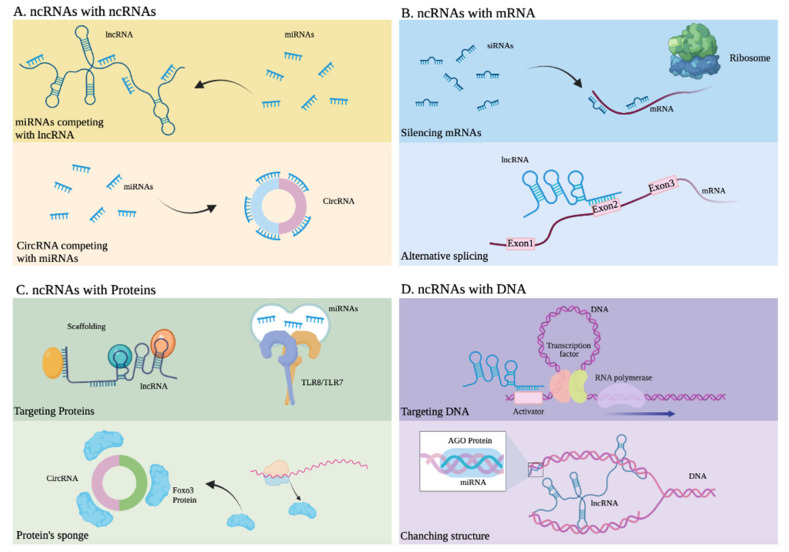
Interactions between noncoding RNAs and other molecules. (**A**) ncRNAs with other ncRNAs. Upper: miRNAs competing with lncRNA. Lower: CircRNA competing with miRNAs. (**B**) ncRNAs with mRNA. Upper: siRNAs silencing mRNA. Lower: Alternative splicing of mRNA due to an lncRNA. (**C**) ncRNAs with proteins. Upper: An lncRNA developing scaffold function and miRNAs activating Toll-like receptors. Lower: A circRNA serving as a sponge or Foxo3 protein. (**D**) ncRNAs with DNA. Upper: An lncRNA targeting the activator of a gene. Lower: An lncRNA altering the structure of DNA.

Many different mechanisms that regulate gene expression (Figure 2), such as transcription factors accessing DNA, and variations in the rates of mRNA synthesis, processing, stability, and translation, are influenced by ncRNAs [58,64]. This is achieved by ncRNAs’ ability to interact with various biological molecules (Figure 3) within different cells and tissues. Among these ncRNAs with regulatory functions, this review particularly focuses on miRNAs and lncRNAs, which have attracted great interest given their roles in various biological functions [51,65,66,67].

miRNAs are generated in the nucleus as pre-miRNAs and processed and exported to the cytoplasm by exportin 5, where they can regulate gene expression mainly at the post-transcriptional level (Figure 3A,C) by binding with other RNAs for regulatory purposes [68,69,70]. Through this mechanism, miRNAs can decrease the expression of certain proteins, through sequence complementarity with their corresponding messenger RNA or through interaction with regulatory lncRNAs. This type of regulation, mainly associated with translational repression, is the mechanism reported in most studies [71,72], along with the regulation of mRNAs through their interaction with lncRNAs. A well-documented example of a regulatory mechanism mediated by miRNAs, which function as modulator of transcripts of coding genes or their regulatory lncRNAs, involves PTEN, a tumor-suppressor gene under complex regulatory control by ncRNA [23,73]. The presence of mature miRNAs within the nucleus has also been reported; these miRNAs activate or silence genes through various mechanisms and, as a result of their direct interaction with DNA or through protein scaffolds (Figure 3D), mechanisms that include epigenetic pathways [74,75,76].

lncRNAs are characterized by their wide functional versatility because they promote the regulation of gene expression either in the nucleus or in the cytoplasm and at different levels transcriptionally and post-transcriptionally, thanks to their ability to interact with other nucleic acids such as DNA and RNA, as well as proteins (Figure 3) [77,78]. In the nucleus, they regulate gene expression in various ways. These include epigenetic modifications by directly binding to DNA and the recruitment of chromatin modifiers. This can lead to a change in the accessibility of genes to DNA-binding proteins, such as transcription factors and even RNA Pol II (Figure 3D), resulting in the activation or suppression of transcription [79,80,81,82]. Another of the most widely studied regulatory mechanisms of lncRNAs involves them acting like enhancers, in which they function either by directly interacting with promoter regions of the genes they regulate or by binding to proteins that participate as mediators [11,83,84,85,86]. These lncRNAs, through their interactions with proteins, recruit such proteins to participate in DNA repair [87]. In the nucleus, these lncRNAs can interact with mRNAs to stabilize them or to direct splicing (Figure 3B) towards a specific mRNA isoform [88,89,90]. Meanwhile, in the cytoplasm, lncRNAs display equally versatile functions. They interact with other ncRNAs such as miRNAs, or with mRNAs or proteins, through mechanisms that can result in the suppression or promotion of the products of the genes that they regulate. For example, some lncRNAs and circular RNAs regulate the activity of miRNAs because they have binding sites that retain them, thus modulating the activity of miRNAs. LncRNAs, which present this mechanism, are considered miRNA sponges, and they are part of a complex interaction network in the transcriptome or also called the theory of competitive endogenous RNAs. Then, miRNA sponges are considered exogenous when they are artificially introduced into a biological system or endogenous when they are expressed naturally [91,92,93]. In addition, ncRNAs, through various interactions with other biological molecules, can be key participants in signaling pathways [94,95,96,97,98,99].

### 2.2. The Importance of Prediction Models That Can Later Be Tested Experimentally

Recently developed laboratory-based techniques have generated major advances in the study of the interactions of RNAs with other biomolecules. These include RAP-RNA, RIA-Seq, hiCLIP, CLASH, PARIS, SPLASH, and LIGR-seq. However, the availability of these approaches is limited, and they are expensive, which impedes study of the ncRNA interactome [100,101].

**Figure 4:**
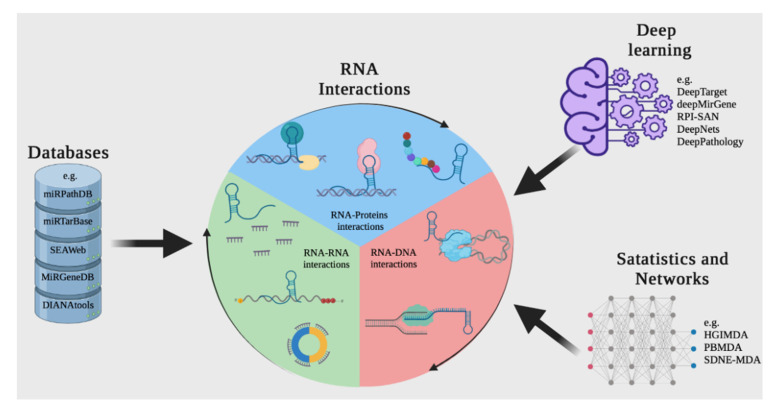
A computational approach for discovering or predicting RNA interactions among different biomolecules. The first strategy is to search in web tools or RNA databases such as miRTarBase. Another way to discover RNA interactions is to use resources based on deep learning and other machine learning-based tools, such as DeepTarget and deepMirGene. Finally, different mathematical and network theory methods can be used to research RNA interactions.

By taking advantage of the information that is generated experimentally using bioinformatics and mathematical algorithms, and storing it in databases and applying open-source tools, it is possible to predict interactions between molecules such as DNA, RNA, and proteins, which can save operating costs and time (Figure 4) [102,103].

The advantage of using ncRNA databases and bioinformatic tools is that, thanks to the fact that they are continually fed new information obtained experimentally and supported by an exhaustive data curation process, they can support the design of experimental trials for targeting and discovering new interactions [104,105].

The various species of RNAs and their products interact in complex ways, the understanding of which provides deeper insights into the functioning of living organisms including humans. This article is intended to boost our understanding of health and disease through clarifying the intricate networks of interactions between heterogeneous biological molecules. Towards this goal, we review the latest advances in databases, bioinformatic tools, and new strategies in silico that allow the establishment or prediction of biological interactions between ncRNAs, particularly miRNAs and lncRNAs. However, these tools also have contained biomolecule-related information in another species.

## 3. Overview of Available Methods for Reconstructing Interactions between ncRNAs and Other Molecules

In this section, we present resources and methods for the reconstruction, analysis, and prediction of interactions between ncRNAs and other molecules (e.g., genes and proteins). Four main approaches are available for focusing on these interactions: (1) obtaining available information about interactions from databases; (2) predicting interactions based on the integration of data available in databases; (3) deep learning methods for analyzing ncRNA interactions; and (4) identifying interactions by analyzing genomic data reporting on ncRNA, mRNA, and/or protein expression.

### 3.1. Databases

In recent years, several databases containing information about the interactions of ncRNAs/lncRNAs/miRNAs with genes or proteins have appeared [106]. They allow information about bipartite interactions to be obtained. A nonexhaustive overview of the most relevant ones is presented in Table S1. Most of them are based on automatic or manual text mining of reported interactions in the scientific literature. Some of them include additional information such as sequences (MirGeneDB, NPInter v4.0), while others also include computational predictions (oRNAment, RNAInter, miRDB, NPInter v4.0, ENCORI).

- DIANA-LncBase

DIANA-LncBase v3.0 is a wide repository of the DIANA Tools initiative. This database contains data on more than 200,000 miRNA–lncRNA interactions based on experimental data from humans and mice. The most recent release was in 2019. DIANA Tools also has applications about other molecules, such as mRNA, DNA, and transcription factors. The DIANA-LncBase database uses an algorithm approach in ~300,000 throughput CLIP-seq datasets to analyze AGO binding events [107,108].

- LnCeVar

LnCeVar was established to provide genomic information about variations in lncRNAs that can affect ceRNA interactions, including SNPs, somatic mutations, and copy number variations. LnCeVar uses curated published information and a dataset of experimental studies. LnCeVar compiles data from TCGA, COSMIC, and the 1000 Genomes Project into a user-friendly interface. It is also possible to download data for analysis and identify and perform visualization of dysregulated variation-ceRNA networks [109,110].

- LncTarD

Li et al. established the newly developed LncTar, a repository with integrative disease–lncRNA–target interactions. It is a manually curated repository that features 2822 interactions in 177 diseases and 475 lncRNAs, based on experimental data. It is claimed that LncTarD understands regulatory networks in the pathogenesis of human diseases [111,112].

- MirGeneDB

MirGeneDB is a previously open-source application, curated and oriented to miRNAs with optimal annotation and nomenclature. The most recent update, MirGeneDB 2.0, includes data on more than 45 organisms, including Homo sapiens and Mus musculus. Together with existing databases including miRCarta and miRBase, it constitutes a robust source for miRNA research. A recently updated web interface allows browsing, searching, and downloading miRNA relevant fasta and annotation files for each organism [113,114].

- miRPathDB 2.0

The novel release 2.0 of the miRNA Pathway Dictionary Database (miRPathDB) holds access to target genes and pathways of all miRNAs from miRBase and miRCarta on humans and mice. It suggests a targetome on miRNAs base, from Integer Linear Program (ILP) development. miRPathDB is linked to other free resources such as miRTarBase, TargetScan, and miRanda [115,116].

- miRTarBase

miRNA–target interactions (MTIs) have attracted particular interest in the scientific community due to the relationship between miRNAs and disease evolution. miRTarBase is an experimentally validated database that obtains information from CLIP-Seq and other high-throughput technology. miRTarBase also integrates databases such as miRBase, SommamiR, miRSponge, and TCGA atlas. miRTarBase aims to provide the most complete collections of validated MTIs for building networks and predicting miRNA interactions [117,118].

- SEAweb

The small-RNA Expression Atlas (SEAweb) is a web application that contains around 4200 sRNA sequence datasets, allowing analysis of published data with Oasis 2, a tool for metadata searching. SEAweb has the advantage of collecting a wide range of datasets of tissue-specific miRNAs across different conditions and dealing with pathologic information to find any association. The ability to download data on differential expression is a feature that allows comparison of one’s own data with information contained in the atlas [119,120].

#### 3.1.1. Prediction Using Computational and Statistical Methods

##### Datasets Making or including Computational Predictions

Some of the databases presented in the preceding section include in silico prediction algorithms for analyzing information found in the scientific literature, combining multiple datasets, and even including sequence information (oRNAment, RNAInter, miRDB, NPInter v4.0, ENCORI) (Table S1).

- oRNAment

The modulation of transcription and translation is essential for homeostasis in cells. It involves robust machinery that continually interacts with RNA and proteins. RNA binding proteins (RBPs) play important roles in the regulation of RNA metabolism and communication with other molecules [121]. To characterize RBP dynamics, scientists at the University of Montreal and McGill University, Canada, developed the oRNAment (oRNA motifs enrichment in transcriptomes) database, which contains the motifs of 223 RBPs experimentally validated by RNAcompete and RBNS platforms. Its main advantage over previous databases is that oRNAment includes putative motifs for RBPs across coding and noncoding transcriptomes in humans, Caenorhabditis elegans, Danio rerio, Drosophila melanogaster, and Mus musculus [122,123].

- NPInter v4.0

NPInter is a database that is already well known. In 2019, it launched a new update with over 600,000 curated interactions. NPInter v4.0 obtains information through text mining and processing experimental data, such as CLIP-seq, PARIS, CLASH, and CHIRP-seq [110]. This database works in two ways: (i) recovering data of GEO and ENCODE and (ii) obtaining information from the RISE database and literature mining. It is intended to find any interactions among ncRNAs and other biomolecules in disease contexts to provide complete detailed annotation and prediction scores [124].

- RNAInter

RNAInter is a database that collects the interactome among heterogeneous biomolecules, with emphasis on protein–RNA interactions. This database uses experimentally obtained and curated data for generated prediction, along with another 35 interaction resources below a unique pipeline. The latest update for this database was released in 2019; it was linked to the RAID v2.0 application, along with RIscoper [125], IntaRNA [126], PRIdictor [127], and DeepBind [128]. RNAInter includes nearly 40 million RNA interactions among 154 species [129].

- ENCORI: The Encyclopedia of RNA Interactomes (StarBase)

ENCORI (The Encyclopedia of RNA Interactomes), previously known as StarBase, is a well-known database that integrates different data among mainly RNA species from high-throughput sequencing studies. It includes data on immunoprecipitated RNAs (CLIP-Seq, HITS-CLIP, PAR-CLIP, CLASH, iCLIP) accompanied by gene expression data on more than 30 cancer types, allowing the design of pan-cancer analyses. ENCORI focuses exclusively on miRNA–ncRNA, miRNA–mRNA, RBP–ncRNA, and RBP–mRNA interactome data for visualization, while also obtaining complementary studies such as those involving survival and differential expression analyses [130,131].

- miRDB

miRDB is a free online database that has improved a computational model for predicting miRNA targets and annotations in five species. This database includes a predicting tool, miRTarget, a support vector machine (SVM) model that was trained with large-scale RNA studies and public CLIP-seq data. miRTarget supplies a probability score calculated by the modeling tool, clarifying the statistical support for the prediction [132,133].

### 3.2. Methods for Predicting Interactions

Another group of tools for studying the interactions between ncRNAs and other molecules uses techniques for the construction of biological networks to integrate different types of data (sequences, structural information, physicochemical properties, etc.). Some of them are reviewed by Zhang et al. (2019). Examples of these include HGIMDA and PBMDA [134].

The method Heterogeneous Graph Inference for MiRNA-Disease Association prediction (HGIMDA) has been developed by Chen et al. (2016) with the aim of predicting potential miRNA–disease associations, that is, genes and proteins participate in diseases, combining different data sources (miRNA functional similarity, disease semantic similarity, Gaussian interaction profile kernel similarity, and experimentally verified miRNA–disease associations) using a graph reconstruction approach [135]. The same group has achieved improvements of HGIMDA, one of which is Path-Based MiRNA-Disease Association (PBMDA, Tokyo, Japan), which integrates three interlinked sub-graphs (i.e., miRNA–miRNA similarity networks, disease–disease similarity networks, and known miRNA–disease association networks) [136].

Another approach proposed by this group is Matrix Decomposition and Heterogeneous Graph Inference for miRNA-disease association prediction (MDHGI) (Chen et al., 2018). This approach achieves improved accuracy by combining two methods (matrix decomposition and networks algorithm) [137].

### 3.3. Deep Learning Methodologies for Genomics

Recently, the implementation of deep learning in the biological sciences has had a great impact on the advancement of omics studies. The basic concepts of deep learning have been derived from artificial neural networks, which mimic the functioning of the human brain to perform complex tasks [138]. Other developments in deep learning are deep structured learning and hierarchical learning, which are useful for making inferences about the quantitative properties of a set of data [139].

In this paper, we do not present a systematic review of the literature. We are instead interested in providing an overview of the currently available deep learning applications for the study of omics data, especially RNA interactions (Table 1).

### 3.4. From Expression Data

Another approach for predicting interactions among ncRNAs, mRNAs, and proteins is based on analysis of the expression levels of these molecules, analogous to transcriptomic analysis. The general concept is to establish similarity measures between data quantifying several types of molecules (i.e., miRNAs and mRNAs) in the same experiment, which indicates the coordinated activity of pairs of the molecules of interest. A threshold to establish a significant level of similarity is then determined. A high value of similarity reflects coordinated activity between the two entities and finally an edge in the co-expression network. If this is applied only for mRNA or protein data, the result is a co-expression network. In this context, it is a bipartite network.

Examples of approaches predicting interactions from expression data are presented by Dragomir et al. [147], Hongbo et al. [148], and Parra-Medina et al. [149].

Dragomir et al. proposed the possibility of constructing a monopartite (miRNAs–miRNAs) co-expression graph using expression levels and a bipartite interaction graph (miRNAs–mRNAs) based on predicted/available association data. In a further step, they proposed an association graph to finally obtain a bipartite graph showing potential associations between miRNAs and mRNAs [147].

Hongbo et al. developed a computational method to identify potential miRNA–disease associations by taking advantage of the functional connections between miRNA targets and disease genes in protein–protein interaction (PPI) networks [148].

Parra-Medina et al. reanalyzed a dataset in which simultaneous miRNA and mRNA expression was available for young and elderly patients to identify differentially expressed miRNAs, from which they constructed an miRNA co-expression network. Through correlation analysis, the hub miRNAs of this network were shown to be related to mRNAs to predict interactions in an indirect manner [149].

## 4. Conclusions

Revealing the interactions between heterogeneous molecules (DNA, RNA, proteins) is a key point to understand cell function. In recent years, information using new generation sequencing methodologies, in the area of ncRNA and other species of RNAs, has been generated and given strength in deepening the knowledge of complex processes such as the modulation of gene expression or the spatiotemporal organization of biomolecules in health and disease.

Molecular interaction experiments, such as HITS-CLIP, PAR-CLIP, iCLIP, PARIS, and SPLASH, among other methodologies, are very expensive. This highlights the importance of applying up-to-date bioinformatics methodologies that interrogate high-quality datasets for the prediction of molecular interactions. Selecting suitable bioinformatics tools depending on the input data, biological question, and available information for the study of interactions between biomolecules is very important.

Here we present a selection of recent tools for this purpose, which can favor the performance of experimental validation of molecular interactions, reducing operating costs. We offer an overview of the state of the art of the different computational applications to complement the study of ncRNA in humans and its potential association with certain diseases such as cancer.

## Figures and Tables

**Table 1:** List of deep learning methodologies in RNomics.

Tool	Approach	Target	Ref.
DeepTarget	Deep recurrent neural network-based auto-encoding and sequence–sequence interaction learning using expression data	miRNA–mRNA interactions	[140]
deepMirGene	Recurrent neural networks (RNNs), specifically long short-term memory (LSTM) networks using expression data	End-to-end learning approach that can identify precursor miRNAs	[141]
RPI-SAN	Auto-encoder neural networks	ncRNA–protein interaction pairs	[142]
DeepNets	Multilayer feed-forward artificial neural networks	RNA-Seq gene expression	[143]
eADAGE	Auto-encoder neural networks	Biological pathway enrichment from expression data	[144]
GCLMI	Graph convolution and auto-encoder	Potential lncRNA–miRNA interactions	[145]
RPITER	Convolution neural network (CNN) and stacked auto-encoder (SAE)	Prediction of ncRNA–protein interactions	[36]
DeePathology	Deep neural networks	Prediction of the origin of mRNA–miRNA interactions	[146]

## Supplementary Materials

The following are available online at https://www.mdpi.com/article/10.3390/ijms222111397/s1.
