# Supplementary material for: Bioinformatic Tools for the Analysis and Prediction of ncRNA Interactions
Source: Int J Mol Sci. 2021 Oct 22;22(21):11397. doi: 10.3390/ijms222111397 (PMC8583695; doi:10.3390/ijms222111397)
Supplement: Supplementary file 1 [file ijms-22-11397-s001.zip › ijms-1397740-supplementary.pdf]

Table S1. ncRNA interactions resources. .

Name	Last update	Number interactions or data	Interaction or scope	Methodology	URL
DIANA-LncBase v3.0	2019	240.000	miRNA-lncRNA	Reporter genes, northern blot, qPCR, RIP-qPCR, biotin miRNA tagging, CLIP-Seq, CLEAR-CLIP, CLIP-chimeric, miR-CLIP, AGO-IP, RNA-Seq, microarrays	http://carolina.imis.athena-innovation.gr/diana_tools/web/index.php?r=site%2Ftools
LnCeVar	2019	119.501 events	Genomic variation in lncRNA	PubMed data curated	http://www.bio-bigdata.net/LnCeVar/
LncTarD	2019	2.822	lncRNA targets (miRNA,mRNA, and protein)	PubMed data curated	http://biocc.hrbmu.edu.cn/LncTarD/ or http://bio-bigdata.hrbmu.edu.cn/LncTarD
MirGeneDB	2019	10.899	miRNA genes	400 publicly available small RNA sequencing datasets	http://mirgenedb.org/
miRPathDB 2.0:	2019	27.452 miRNA candidates 1.978 miRNA interactions	miRNA-mRNA	miRBase (Version 22.1) and from miRCarta (Version 1.1) data	https://mpd.bioinf.uni-sb.de/
miRTarBase	2019	13.404 interactions	ncRNA, mRNA, Transcription factors	RNA-binding proteins (RBPs) and mRNAs from large-scale CLIP-Seq (HITS-CLIP, PAR-CLIP, iCLIP, CLASH) data	http://miRTarBase.cuhk.edu.cn/
SEAweb	2019	4200 published samples	miRNA, piRNA, snoRNA, snRNA, siRNA	Text mining pipeline	http://sea.ims.bio/
ENCORI (starBase)	2014?	>10 millons	miRNA-ncRNA, miRNA-mRNA, ncRNA-RNA, RNA-RNA, RBP-ncRNA, and RBP-mRNA	AGO CLIP Seq, Degradome-seq, RBP CLIP-Seq, RNA-RNA Interactome	http://starbase.sysu.edu.cn/index.php

RAIN	2017 Junge et al (2017)	>10000	Interactions by non-coding RNA (ncRNA)	ncRNA–RNA and ncRNA–protein	https://rth.dk/resources/rain/
**Databases and prediction tools**					
NPInter v4.0	2019	609.242 Interactions	ncRNA-ncRNA ncRNA-DNA	Manual literature mining, processing of high-throughput sequencing data.	http://bigdata.ibp.ac.cn/npinter
oRNAment	2019	223 RBPs, 453 motifs	Putative RBP binding site in both coding and non-coding RNA	Position weight matrices (PWMs)	http://rnabiology.ircm.qc.ca/oRNAment/
miRDB	2020	3,5 millons	miRNA-target interactions	CLIP-ligation data, Support vector machine (SVM) model	http://mirdb.org.
RNAInter (update of RAID)	2019	>41 million RNA-associated interaction	RNA-RNA and RNA-protein RNA-DNA/compound	Experimentally validated and computationally predicted RNA-associated interactions	http://www.rna-society.org/rnainter/ or http://www.rna-society.org/raid/
